# Relationship between Bone Mineral Density and Maturity Index in Cervical Smears, Serum Estradiol Levels and Body Mass Index

**DOI:** 10.5539/gjhs.v5n6p209

**Published:** 2013-09-29

**Authors:** Homayoun Sheikholeslami, Majid Sotodeh, Amir Javadi, Neda Nasirian, Amir Mohammad Kazemifar, Mahnaz Abbasi

**Affiliations:** 1 Qazvin University of Medical Sciences, Qazvin, Iran

**Keywords:** bone mineral density, body mass index, cervical smear, serum estradiol level, maturity index

## Abstract

**Objectives:**

Osteoporosis is a systemic skeletal disease with a consequent increased risk of fracture, decreased quality of life and economic burdens for both the patients and health care system. While Dual energy X-Ray absorptiometry remains the gold standard for assessment of bone mineral density, it cannot be requested for all patients for obvious reasons. By determining other variables that may correlate with osteoporosis, we can identify individuals who may be at risk for osteoporosis earlier. Then, they can be treated at the earlier stages of the disease. In the present study, relationships between bone mineral density, maturity index in cervical smear, serum estradiol level and body mass index were examined.

**Materials & Methods:**

The present study performed on 128 women, who had been referred for bone mineral densitometry. Blood samples were obtained for determination of serum estradiol level. Cervical smear was taken for assessment of cell’s maturity. Cervical smears were examined by a pathologist and were sorted as atrophic or mature. Body mass index was calculated too. Relationships among Body mass index, serum estradiol level, and maturity index and bone mineral densitometry were analyzed using proper statistical tests.

**Results:**

Maturity index had significant relationship with T Score in the spine and femoral neck (P<0.001). Significant relationships were found between serum estradiol level and femoral neck T Score (P<0.004) and spine T Score (P<0.008). Also a significant relationship was found between body mass index and bone mineral density.

**Conclusion:**

Pap smear is a routine examination which is performed mainly for screening purposes in gynecology. It is non-invasive, simple and low-priced. Results of current study suggest that women with atrophic cervical smear should be examined more rigorously for osteoporosis. If any patient has atrophic maturity index in her cervical smear, she will be at much higher risk of osteoporosis.

## 1. Introduction

Osteoporosis is quite common in elderly; particularly women. Osteoporosis is characterized by lessened bone density, lowered bone’s quality, and increased bone fragility. It is associated with risk of pathologic fracture which may adversely affect patient’s quality of life. It’s financial burden on the patient and health system is noticeable. Accordingly, its early diagnosis is crucial.

Prevalence of osteoporosis is going to reach to the point of epidemic; owing to increase in elderly population in societies and unwarranted use of steroids for various diseases. Early diagnosis of osteoporosis makes prevention of its grave complications possible; especially pathologic fracture. The most common diagnostic tool for assessment of bone mineral density (BMD) is bone mineral densitometry; through which osteoporosis and risk of fracture can be diagnosed and response to treatment can be assessed (Jonell et al., 2006; Kanis et al., 2001). At present, dual energy X-ray absorptiometry (DEXA) is the preferred method for bone mass densitometry (Bates et al., 2002).

According to recommendation of the world health organization (WHO) dual energy X-ray absorptiometry (DEXA) should be requested for every menopaused or older than 65 woman, if she has any risk factor for osteoporosis (Kanis et al., 2000). With these criteria, some younger women without related risk factors may suffer from undiagnosed osteoporosis. So, other criteria might also be considered for proper diagnosis of such patients to improve their outcome.

Some authors believe that higher basal metabolic rate (BMR) is associated with lower risk of osteoporosis. Consequently measurement of patient’s height, weight, and BMI can be used as diagnostic variables for osteoporosis (Repse-Fokter et al., 2007). Furthermore, decrease in estradiol or estrogen levels can aggravate the rate of cortical bone loss; both before and after menopause (Gennari et al., 2005). On the other hand, low estrogen level may prevent maturation of vaginal epithelial cells which can be seen in cervical smear as atrophic cells and lowered maturity index (Gennari et al., 2005). Previous reports have mentioned that osteopenia and osteoporosis are more prevalent in women with atrophic vaginal cells. They have suggested DEXA for such patients; even if they have apparently low probability of osteoporosis and low BMD indices (Gennari et al., 2005).

If a sample from upper lateral wall of vagina is taken during routine Pap smear, it can be assessed for estrogen effect using a test named maturity index. Pap smear is widely used as screening test during gynecologic examination. If any association between maturity index and osteoporosis is present, the result of pap smear can be used for assessment of the risk of osteoporosis, especially in low risk women. Present study was designed to evaluate relationship between BMD (using DEXA method) and serum estradiol concentration, maturity index in cervical smear, and BMI.

## 2. Materials and Methods

Present study was conducted on 128 patients who had been referred for bone mass densitometry (DEXA method) to a clinic by their physicians. They had been given informed consent before participation in the study. The study has been approved by local ethical committee of Qazvin’s University of medical sciences. Iran. Their BMI was determined by one of the authors. Peripheral blood sample was taken from their ante-cubital fossa for determination of serum estradiol concentration. Cervical smear was also taken from them at the same time. The samples were taken at the time of referral for menopaused women, or another time during their second half of menstrual cycle for non-menopaused women. If any patients had been used any hormone, contraceptive pill, corticosteroid, or any vaginal cream during previous 1 month, she was excluded from the study.

Cervical smears were examined by a one of the authors who is pathologist. They were categorized as atrophic pattern and mature pattern according to their maturity index. Maturity index was determined by counting parabasal, superficial, and intermediate cells during microscopic examination of 100 random cells in cervical smear. Maturation of parabasal cells are not affected by estrogen or progesterone. Intermediate cells mature under influence of progesterone. Superficial cells maturation is affected by estrogen. Maturity index changes along with menstrual cycle too. Cervical smear will be regarded “atrophic”, if more parabasal cells are seen in it. While, it will be considered “mature”, if superficial cells are the predominant cell seen. Intermediate cells have little clinical implication (McEndree, 1999).

Serum estradiol concentration was determined using radioimmunoassay (RIA) method (Lee et al., 2006). The commercial kits used were obtained from Beckman Coulter Company, England which use anti –estradiol coated tubes with I_125_ labeled estradiol in ethanol. The normal level was considered less than 82 pg/ml for menopaused women and 71-277 pg/ml for non-menopaused women in middle-luteal phase.

The patients were sorted into normal, osteoporotic, and osteopenic based on result of their BMD. Then distribution of BMI, maturity index, and serum estradiol concentration was evaluated in them. Ki-square, exact Fischer, and correlation tests were used for statistical analysis of resulting data, whatever was relevant.

## 3. Results

One hundred twenty eight patients were examined in the current study. 44 patients were non-menopaused and 84 were menopaused. Their mean ages were 45.5±9 years and 58.7±7 years in non-menopaused and menopaused women respectively. Their BMIs were 29.5±4.7 kg/m^2^ and 26.8±5.1 kg/m^2^ in non-menopaused and menopaused patients respectively. Results of BMD at vertebral column and femoral neck are illustrated in tables 1 and 2.

**Table 1:** Results of bone mass densitometry of spinal column (normal, osteopenia, and osteoporosis) in menopaused and non-menopaused patients

	Osteoporosis	Osteopenia	Normal	Total
Menopaused	21 (25%)	30 (35.7%)	33 (39.3%)	**84**
Non-Menopaused	1 (2.3%)	8 (18.2%)	35 (79.5%)	**44**

**Table 2:** Results of bone mass densitometry of femoral neck (normal, osteopenia, and osteoporosis) in menopaused and non-menopaused patients

	Osteoporosis	Osteopenia	Normal	Total
Menopaused	25 (29.8%)	25 (29.8%)	34 (40.4%)	**84**
Non-Menopaused	3 (6.8%)	8 (18.2%)	33 (75%)	**44**

29.5% of non-menopaused women and 77.4% of menopaused women had atrophic maturity index in their cervical smear. As it can be seen in Table 3, patients with atrophic maturity index more frequently had osteoporosis or osteopenia in their vertebral column. The difference was statistically significant (p-value less than 0.001). The same is true for femoral neck (p-value less than 0.001). The details are demonstrated in Table 4. According to these results, any patient with atrophic cervical smear has 42.5 and 22.9 times higher risks of developing osteoporosis or osteopenia in vertebral column and femoral neck respectively, compared to woman with mature smear.

**Table 3:** Frequency distribution of maturity index of cervical smear in patients with normal, osteopenia, and osteoporosis in their BMD of spinal column

	Osteoporosis	Osteopenia	Normal	Total
Mature	0	3 (6%)	47 (94%)	**50**
Atrophic	22 (28.2%)	35 (44.9%)	21 (26.9%)	**78**

**Table 4:** Frequency distribution of maturity index of cervical smear in patients with normal, osteopenia, and osteoporosis in their BMD of femoral neck

	Osteoporosis	Osteopenia	Normal	Total
Mature	0	5 (10%)	45 (90%)	**50**
Atrophic	28 (35.9%)	28 (35.9%)	22 (28.2%)	**78**

What’s more, BMI had statistically significant relationship with results of BMD of vertebral column and femoral neck (p-value less than 0.001). We calculated that any person with BMI higher than 30 kg/m^2^ had 77% and 78% less chances of developing osteoporosis or osteopenia in vertebral column and femoral neck respectively, compared to patients with BMI less than 29.9 kg/m^2^.

Moreover, Serum estradiol concentration had statistically significant relationship with of BMD of vertebral column and femoral neck (p-values less than 0.004 and 0.008 respectively). However, their corresponding r- values were 0.22 and 0.25. It means serum estradiol concentration can predict T-score changes of bone mass densitometry of vertebral column and femoral neck only in 5 and 6 percent of the patients, respectively. So, the correlations were rather weak.

## 4. Discussion

In present study, we found significant relationship between maturity index in cervical smear and results of bone mass densitometry of vertebral column and femoral neck. Furthermore, our findings showed relationship between BMI and results of BMD too.

It is expected that prevalence of osteoporosis will increase, as a result of increase of elderly population in our country. Osteoporosis might complicate with pathologic fracture which has health and economic burdens for patients, his/her family, and society. Its prevention through its early diagnosis is crucial.

Many studies were conducted to suggest a simple and effective method for early diagnosis of osteoporosis (Bates et al., 2002; Repse-Fokter et al., 2007; Gennari et al., 2005; De Laet et al., 2005; Reps-Fokter et al., 2003). BMI has been suggested for this purpose (De Laet et al., 2005). Present study found negative relationship between BMI and BMD. We found that BMI less than 29.9 kg/m^2^ is a risk factor for development of osteoporosis. This is in agreement with related studies (Repse-Fokter et al., De Laet et al., 2005). For instance, Fawzy and his coworkers (2011) have found statistically significant correlation between BMD and BMI in their study on 101 subjects. They have suggested low BMI as a major risk factor for low BMD.

We also found relationship between serum estradiol concentration and results of bone mass densitometry. Though there was not significant correlation between them. Possibly, if the sample size was larger or blood sampling was performed in certain day of menstrual cycle, more decisive relationship would be reached. The relationship between estradiol concentration and BMD has been notable in some reports. Mawi (2010) has conducted a study on 184 menopausal women. He reported that serum estradiol concentration higher than 5 pg/ml has statistically significant relationship with T-score in bone mass densitometry of femoral neck. Study of Bagur and his colleagues has found that if serum estradiol concentration was higher than10 pg/ml in menopausal women, their scores would be higher in bone mass densitometry all parts of the body (Bagur et al., 2004). The same results have also been reported in other study (Van Geel et al., 2009).

Pap smear is a routine examination which is performed mainly for screening purposes in gynecology. It is non-invasive, simple and low-priced. Results of current study suggest that women with atrophic cervical smear should be monitored more meticulously for osteoporosis. We found that if any patient has atrophic maturity index in her cervical smear, she will be at much higher risk of osteoporosis, when compared to patient with mature maturity index.

Fokter and his coworkers (2003) have studied morphologic characteristics of squamous cells in cervical smear and its relationship with mineral density of bone. They have determined nucleus to cytoplasm diameters ratio (N/C ratio) in the cells using electron microscopy. They found that higher N/C ratio means atrophy of squamous cells and has relationship with bone density in femur and vertebral column. Present study found similar relationship. However, we used light microscopy instead of electron microscopy, which is more accessible.

With the best knowledge of the authors, no similar study has been published about maturity index cervical smear and its relationship with osteoporosis, up till now. Consequently, we cannot compare results of present study with similar studies. We recommend similar studies are designed in other centers to evaluate the relationship between cervical smear for screening of osteoporosis.
